# Pessary management practices for pelvic organ prolapse among Australian health care practitioners: a cross-sectional study

**DOI:** 10.1007/s00192-023-05540-2

**Published:** 2023-05-24

**Authors:** Katrina McEvoy, Rebecca Griffin, Melissa Harris, Hannah Moger, Olivia Wright, Irena Nurkic, Judith Thompson, Rebekah Das, Patricia Neumann

**Affiliations:** 1 Curtin University, Bentley, WA Australia; 2 University of South Australia, Adelaide, SA Australia

**Keywords:** Health care practitioner, Pelvic health, Pelvic organ prolapse, Vaginal support pessary, Survey, Multidisciplinary

## Abstract

**Introduction and hypothesis:**

Vaginal pessaries are a low-cost, effective treatment for pelvic organ prolapse (POP) and an alternative to surgery. Whilst traditionally pessary management (PM) has been provided by medical professionals, particularly gynaecologists, recent international studies found other professionals, including physiotherapists and nurses, may be involved. It is unknown which health care practitioners (HCPs) provide PM for POP in Australia or the distribution of services.

**Methods:**

In a cross-sectional study design, a self-reported electronic survey investigated Australian HCPs providing PM for POP. Purposive and snowball sampling targeted HCPs, professional organisations and health care facilities. Descriptive statistics described PM in relation to HCP professional profile, PM provision and geographical location.

**Results:**

There were 536 respondents (324 physiotherapists, 148 specialists, 33 general practitioners (GPs) and 31 nurses providing PM. Most worked within metropolitan regions (*n* = 332, 64%), 140 (27%) in rural, 108 (21%) in regional and 10 (2%) in remote areas. Most worked privately (*n* = 418, 85%), 153 (46%) worked publicly and 85 (17%) in both. Ring pessaries were most commonly used, followed by cube and Gellhorn. HCPs reported variable training in PM, and 336 (69%) had no mandatory workplace competency standard; however, 324 (67%) wanted further training. Women travelled long distances to access services.

**Conclusions:**

Doctors, nurses and physiotherapists provided PM in Australia. HCPs had variable training and experience in PM, with rural and remote HCPs particularly wanting further training. This study highlights the need for accessible PM services, standardised and competency-based training for HCPs, and governance structures ensuring safe care.

**Supplementary information:**

The online version contains supplementary material available at 10.1007/s00192-023-05540-2.

## Introduction

Pelvic organ prolapse (POP) is a prevalent condition that can impact a woman’s quality of life (QoL). POP refers to the descent of one or more of the vaginal walls, uterus, or apex of the vagina [1]. The most reported symptom of POP is vaginal bulge, which is often accompanied by bladder, bowel, and/or sexual dysfunction [1]. Based on the current Australian population [2], with a prevalence of 5–10% [3], an estimated 472,000 to 945,000 women are symptomatic and potentially require access to health services for management of POP [4].

Treatment options for POP include surgery, pelvic floor muscle training, lifestyle advice and vaginal pessaries [5]. Up to one in five women will require POP surgery before age 80 [6], with recurrent prolapse common [7].

A pessary is a cost-effective device used within the vagina to provide support to the vaginal walls and prolapsed organs [8, 9]. Pessaries may be as effective as surgery in improving QoL, prolapse and urinary symptoms [10]. However, they have the potential to cause complications ranging from vaginal discharge and erosions to fistula formation, sepsis, and rarely, death [11]. Complications can be mitigated by ensuring correct pessary fitting and sizing, thorough client education and appropriate follow-up, which requires trained and competent practitioners [11].

Traditionally PM has been provided by gynaecologists, however recent international surveys have found other professionals may also be involved [12–17] working across various clinical settings, often without clinical practice guidelines or standardised training requirements [13, 14, 18]. One Australian study described a 1-day multi-disciplinary training program [19], but there are no data on Australian pessary providers or services.

International studies on PM found the majority of surveyed HCPs in the United Kingdom (UK) and France worked within major hospitals or the National Health Service (NHS) [12, 13, 15, 20]. However, the Australian population distribution and health care system differ, and whilst Medicare provides universal healthcare, 56% of the population have private health insurance. Women may, therefore, seek PM from public or private clinics [21]. Furthermore, 28% of Australians live in rural and remote areas [22], while specialised hospital services are predominantly located in major centres around the coast. This presents unique geographical challenges to health service delivery and women’s access to high-quality services to manage their prolapse in the way they choose. Hence, information is needed about pessary services, their location in urban and rural areas, and HCPs’ training in PM, with a view to informing future service and workforce educational needs. This information is particularly important given the evidence of poorer health outcomes for people in rural areas [22].

## Aims

The aims of this study were to establish which HCPs are providing PM for POP in Australia and where these services are delivered. Secondary aims were to investigate the professional profile of the HCPs and the types of pessaries used.

## Materials and methods

In this cross-sectional study, an anonymous 24-item electronic survey (Electronic Supplementary Material) was distributed to HCPs providing PM for POP. Ethical approval was granted by Curtin University Human Research Ethics Committee (HREC) (HREC number HRE2022-0325).

The survey was developed by co-investigators with experience in PM, using concepts from similar international studies [12, 13, 17, 20, 23] and clinical practice guidelines [5, 24]. Representatives from each target discipline (medical, nursing, and physiotherapy) and the Urogynaecological Society of Australasia (UGSA) were invited to complete the draft questionnaire and provide comment to ensure relevance and usability. Feedback provided was incorporated into the final design. The survey contained both multiple choice and text answer questions, divided into four sections: clinician demographics, PM training, current PM practices and future training needs. HCPs were asked to report their geographical location in relation to the Modified Monash (MM) model [25]. MM categories range from MM1 (major cities) to MM7 (very remote communities) and are based on population size and remoteness of each area. For geographical location reporting, MM1 will be subsequently referred to as 'metropolitan’, MM2 as ‘regional’, MM3–MM5 as ‘rural’ and MM6–MM7 as ‘remote’.

The survey was distributed using the Qualtrics XM (Provo, UT) platform, between 26 June and 31 August 2022 via a QR code and web link. Participant consent was required via a checkbox at the commencement of the questionnaire. To optimise responses, associations willing to send reminder emails were asked to do so 14 days after initial distribution, and participant burden was minimized with a time-efficient (5-minute, author-trialled) survey, containing branching questions and an option to save and return. Forced responses avoided missing data.

Recruitment targeted HCPs providing PM in a range of Australian health care settings. Target respondents were identified as medical specialists (urogynaecologists, obstetrician–gynaecologists, gynaecologists, GP–obstetricians, urologists), GPs, physiotherapists and nurses. GP responses were analysed separately from other medical respondents due to their unique role in primary care. GPs provide referrals to specialists and co-manage pessaries together with physiotherapists, whose scope of practice in Australia precludes diagnosis of pathology and laboratory testing.

Organisations who agreed to distribute the survey to their members were UGSA, Australian College of Rural & Remote Medicine, Australasian Gynaecological Endoscopy & Surgery Society, Rural Doctors Association, Continence Nurses Society of Australia, Continence Foundation of Australia and Australian Physiotherapy Association (APA) Women’s, Men’s and Pelvic Health Group. We contacted relevant public health facilities, hospital women’s/pelvic health clinical networks, unit managers of known public health pessary clinics, continence clinics and remote continence services, and known physiotherapy pessary/women’s health education and training networks. Pessary suppliers were contacted and asked to distribute the survey to HCPs or professional organisations purchasing pessaries. Recruitment amongst professional contacts also occurred via email, flyers and social media advertising. As there were no existing data on pessary services or providers, an ideal sample size could not be estimated. Furthermore, the snowball and purposive sampling methods also prohibited calculation of a response rate, as the total number of people who viewed recruitment material versus completed the survey could not be known.

Data were exported to Excel in csv format for cleaning before importation into Jamovi 2.3 for statistical analysis. Missing data were accounted for and results from partially completed surveys were included in statistical analysis. Response attrition was observed within 54 surveys. Calculations relating to percentages were adjusted to reflect the individual question response rate.

Responses were analysed using descriptive statistics and key variables were reported using frequencies (numbers and percentages), including HCP professional profile, geographical location of PM services, clinical settings, aspects of PM and training. Descriptive comparisons were made between professions for years in profession, years fitting pessaries, number of pessaries fitted per month, geographical areas, clinical settings, pessary types used, age groups of patients managed with pessaries, completed training and desire for future training. Respondents who agreed that patients travelled long distances to access pessary services were analysed per geographical area. Finally, a density map was created to demonstrate the distribution of HCPs across Australia. To protect anonymity, data from a single geriatrician respondent was excluded from tables.

## Results

In total, 568 responses were obtained. Responses from an unqualified medical student (*n* = 1) and participants who did not proceed beyond question one (relating to professional qualification) (*n* = 31) were excluded, leaving 536 responses for analysis.

### HCP providing PM

Demographics of respondent HCPs are outlined in Table 1. Respondents were predominantly physiotherapists, followed by obstetrician–gynaecologists, gynaecologists, GPs, nurses and urogynaecologists. Nurses, GPs and GP–obstetricians reported the longest professional experience. Years of PM experience varied, with specialists reporting on average (median) 12 and 13 more years of experience compared to physiotherapists and nurses respectively. Nurses and urogynaecologists recorded the most pessaries fitted per month.

**Table 1:** Demographics of HCP respondents by professional group (*N*= 536)

	Total respondents *n* (%)	Years qualified median (IQR)	Years of PM median (IQR)	Ave pessaries per month median (IQR)	Further training desired: *N* = 482* *n* (%)
Specialist (total)	148 (28%)	16.0 (18.0)	17.0 (15.5)	5.0 (7)	46 (34%)
OB–GYN / OB	59 (11%)	14.0 (15.0)	17.0 (12.5)	4.0 (3.5)	21 (38%)
UROGYN	28 (5%)	16.0 (21.0)	20.0 (20.0)	8.0 (9.5)	0
GYN	44 (8%)	20.0 (19.8)	20.0 (15.3)	5.0 (8.0)	14 (34%)
Urologist	8 (1%)	10.5 (7.7)	10.0 (6.3)	4.5 (1.0)	4 (50%)
GP–OB	8 (1%)	21.5 (32)	12.0 (24.8)	2.0 (1.5)	6 (85%)
GP	33 (6%)	22.0 (27.0)	10.0 (16.8)	3.0 (1.5)	16 (67%)
Physiotherapist	324 (60%)	14.5 (13.0)	4.0 (4.0)	3.0 (3.5)	244 (82%)
Nurse	31 (6%)	20.0 (26)	5.0 (8.0)	11.0 (26.3)	18 (75%)

* N = 482 responses to this question

PM = pessary management, OB–GYN = obstetrician–gynaecologist; OB = obstetrician; UROGYN = urogynaecologist; GYN = gynaecologist; GP–OB = general practitioner–obstetrician; GP = general practitioner

A general practitioner–obstetrician (GP–OB) is a general practitioner with additional training in obstetrics, providing obstetric care particularly in rural and remote Australia

### Geographical location of pessary services

**Fig. 1:**
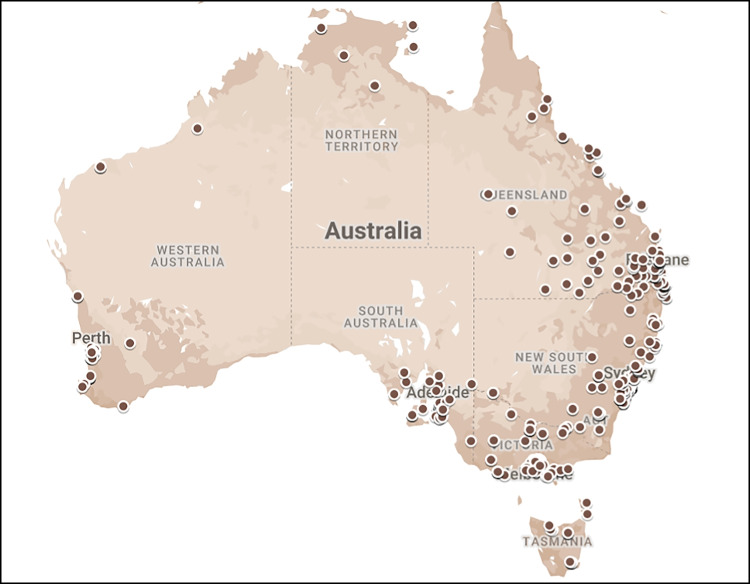
Distribution of HCP respondents

Respondents were predominantly located within metropolitan areas (*n *= 332, 64%) (Fig. 1). One-hundred and eight respondents (28%) practiced within regional centres, 140 (27%) in rural regions, and 10 (2%) in remote regions. More HCPs in rural (*n *= 106, 83%) and remote areas (*n *= 8, 80%) reported that patients travelled long distances for PM compared to those in regional centres (*n *= 74, 70%) and in metropolitan areas (*n *= 175, 55%).

### Pessary service provision

Table 2 displays the distribution of HCPs across public and private health care settings. Four hundred and eighteen respondents (85%) worked in private settings and of those in private clinics, 149 (36%) were the sole provider of PM. One hundred and fifty-three HCPs (31%) worked publicly with 70 (46%) of these working exclusively in the public sector. Urogynaecologists and nurses were the most common HCP within public pessary clinics and public hospital outpatients.

**Table 2:** Mode of service delivery amongst HCP (*N* = 488)

	Public hospital inpatient	Public hospital outpatient	Public pessary clinic	Public community & rural outreach	Private hospital	Private practice (1 PM provider)	Private practice (> 1 PM provider)
All HCPs (*N* = 488) (%)	*n* = 30 (6%)	*n* = 139 (28%)	*n* = 27 (6%)	*n* = 22 (4%)	*n* = 39 (8%)	*n* = 221 (45%)	*n* = 225 (46%)
Specialist (*n* = 137) (%)	*n* = 27 (20%)	*n* = 72 (52%)	*n* = 14 (10%)	*n* = 11 (8%)	*n* = 34 (25%)	*n* = 73 (53%)	*n* = 51 (37%)
OB–GYN & OB (*n* = 57) (%)	*n* = 8 (14%)	*n* = 21 (37%)	*n* = 3 (5%)	*n* = 3 (5%)	*n* = 10 (18%)	*n* = 31 (54%)	*n* = 18 (32%)
UROGYN (*n* = 24) (%)	*n* = 18 (75%)	*n* = 24 (100%)	*n* = 10 (42%)	*n* = 2 (8%)	*n* = 12 (50%)	*n* = 16 (67%)	*n* = 5 (21%)
GYN (*n* = 41) (%)	*n* = 7 (15%)	*n* = 22 (54%)	*n* = 1 (2%)	*n* = 1 (2%)	*n* = 9 (22%)	*n* = 19 (46%)	*n* = 19 (46%)
Urologist (n=8) (%)	*n* = 1 (12%)	*n* = 4 (50%)	*n* = 0 (0%)	*n* = 1 (13%)	*n* = 3 (38%)	*n* = 4 (50%)	*n* = 4 (50%)
GP–OB (*n* = 7) (%)	*n* = 1 (14%)	*n* = 0 (0%)	*n* = 0 (0%)	*n* = 2 (29%)	*n* = 0 (0%)	*n* = 3 (43%)	*n* = 5 (71%)
GP (*n* = 23) (%)	*n* = 0 (0%)	*n* = 2 (9%)	*n* = 0 (0%)	*n* = 2 (9%)	*n* = 0 (0%)	*n* = 8 (35%)	*n* = 12 (52%)
Physiotherapist (*n* = 303) (%)	*n* = 0 (0%)	*n* = 47 (15%)	*n* = 4 (1%)	*n* = 8 (3%)	*n* = 4 (1%)	*n* = 140 (46%)	*n* = 153 (50%)
Nurse (*n* = 25) (%)	*n* = 3 (12%)	*n* = 18 (72%)	*n* = 9 (36%)	*n* = 1 (4%)	*n* = 1 (4%)	*n* = 0 (0%)	*n* = 9 (36%)

OB–GYN = obstetrician–gynaecologist; OB = obstetrician; UROGYN = urogynaecologist; GYN = gynaecologist; GP–OB = general practitioner–obstetrician; GP = general practitioner, PM = pessary management

Note % may exceed 100% as multiple responses were allowed

Three hundred and thirty-six respondents (69%), predominantly those in private hospitals and private practices, indicated that there was no competency requirement in their workplace.

**Fig. 2:**
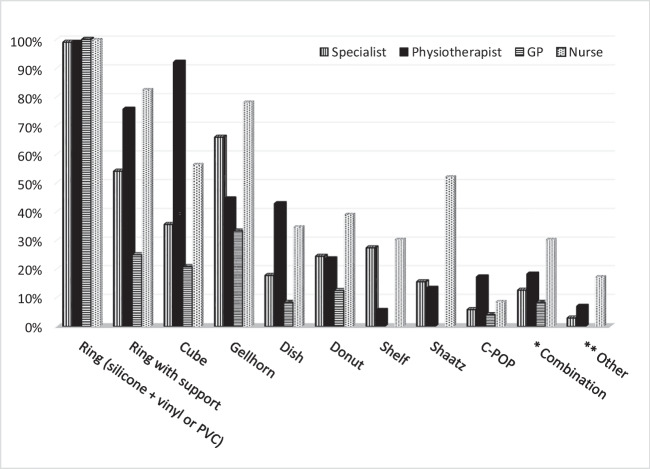
Pessary types used in clinical practice by professional group

Figure 2 displays the pessary types reported. The majority of respondents (*n* = 482, 99%) reported using ring pessaries, with 325 (67%) of these using silicone rings, 45 (9%) using PVC or vinyl rings, and 110 (23%) using both silicone and vinyl. Specialists, nurses and GPs predominantly used Gellhorns as space-occupying pessaries, whereas physiotherapists used cubes. Nurses recorded the highest use of combination and ‘other’ pessary types.

When identifying their most commonly fitted pessary type, 424 HCPs (90%) selected ring (silicone, vinyl, or PVC), 24 (5%) cube pessaries, and 12 (3%) selected Gellhorn. Fifty HCPs (10%) did not fit a second type of pessary, and 153 (32%) did not fit a third.

**Fig. 3:**
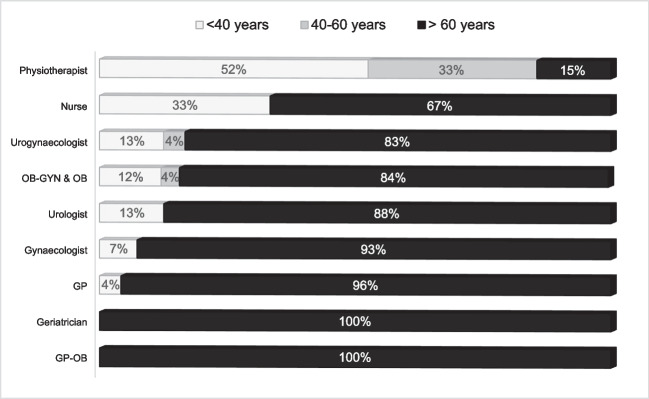
Most common age group of women fitted with pessaries per profession

Medical and nursing professions fitted pessaries most commonly for women > 60 years of age, whereas 85% of physiotherapists most commonly fitted pessaries for women < 60 years of age (Fig. 3).

The majority of HCPs (*n* = 458, 94%) provided all aspects of PM: pessary sizing and fitting, follow-up and changing pessaries, and teaching self-management. Thirty-one HCPs (6%) did not size and fit pessaries, including 14 GPs (58%) and eight nurses (33%).

### Training in PM

**Fig. 4:**
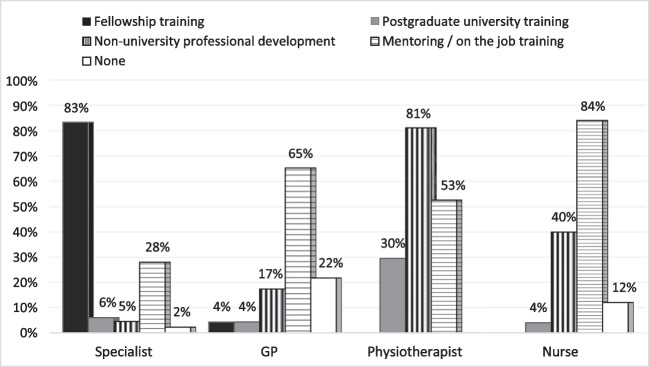
Pessary training type per profession

There were 471 responses to the PM training question, displayed in Fig. 4. Specialists (*n* = 132) were predominantly trained within their fellowship program (*n* = 110, 83%), nurses (*n* = 25) and GPs (*n* = 23) predominantly learnt through mentoring/on the job training (84% and 65% respectively). Physiotherapists (*n* = 291) received training in PM through non-university professional development (*n* = 236, 81%) and postgraduate university training (*n* = 86, 30%). Mentoring/on the job training was reported by 226 HCPs (48%), with 44 of those (9%), predominantly GPs and nurses, indicating that mentoring was their only form of training. Eleven respondents (2%), including specialists, GPs and nurses, reported no PM training.

As indicated in Table 1, 324 (67%) of respondents wanted further training in PM, the majority in complex case and adverse event management, followed by risk mitigation, sizing and fitting of other pessaries, skill development and mentoring others, and service management. Twelve HCPs (100%) working in remote areas wanted further training, in comparison to 133 rural HCPs (75%) and 66 regional HCPs (64%) and 202 metropolitan HCPs (65%).

## Discussion

To our knowledge, this is the first study to investigate PM services in Australia. Our findings are consistent with international literature, demonstrating that pessary services are delivered by a range of professional groups. However, we found that physiotherapists were the most common respondents (60%), contrasting with international studies, which identified doctors [12], specifically, gynaecologists [15], nurses [13], and combined teams of doctors and nurses [20] as the most common. Previously, the first report of physiotherapists providing PM was a 2013 UK study which surveyed professional associations to investigate pessary provision for POP and urinary incontinence [12]. Of the 527 respondents, 487 (96.8%) were doctors, nine (1.8%) were nurses and only seven (1.4%) were physiotherapists [12]. More recently, in a UK survey, Brown et al reported that of 403 respondents involved in PM, 246 (61%) were nurses, 134 (33%) were doctors, and 22 (5%) were physiotherapists [13], while Pizzoferrato [15] reported that 208 (20.4%) respondents to a similar French survey were physiotherapists. The high representation of physiotherapists in our study may reflect the fact that, in the last decade, as pessary training has become available for physiotherapists in Australia, Women’s Health physiotherapists have increasingly embraced PM as part of holistic conservative prolapse management. Also, as physiotherapy researchers, it is likely that we have been able to recruit most Australian physiotherapists providing PM. Physiotherapists reported variable training in PM, with the majority (81%) trained in non-university-based professional development courses, while 30% had competency-based postgraduate PM training.

We received 103 responses from gynaecologists and obstetrician–gynaecologists, representing only 4.7% of those registered in Australia [26]. Similarly, only 33 GPs responded to our survey, representing < 0.1% of those registered [26]. Whilst the number of obstetrician–gynaecologists and GPs involved in PM is unknown, we suspect under-representation in our results. Nonetheless, the low numbers of GPs possibly providing PM is of concern due to their important primary-care role in Australia, providing PM for women with uncomplicated prolapse and co-managing these women with appropriately trained physiotherapists. It has been suggested that the provision of PM in tertiary settings is not cost-efficient or convenient for patients [27], while others have advocated for increased HCP education to enable more PM in primary care settings [28]. This may be of particular relevance in Australia, where women in rural and remote areas have limited access to specialist services and must rely on local GP services [29].

Results from our study indicate PM occurs in public and private settings across Australia. Eighty-five percent of survey respondents worked in the private sector. However, our data is skewed by the high number of physiotherapy respondents who work primarily in private practices. This finding contrasts with studies from the UK, where pessary services were mostly delivered within public sector multidisciplinary teams and few HCPs reported working privately [13, 20]. Similarly, a study of gynaecologists from the Netherlands reported PM being provided predominantly in hospitals, with only 1% in private practice [16]. Our survey respondents were mainly in metropolitan areas, where the density of HCPs is greater across all professions compared to regional and remote areas [29]. Twenty-one percent of respondents worked in regional centres, 27% in rural areas, and only 2% in remote areas. The percentage of respondents in each geographical location is similar to the population density of Australians living in these areas, with numbers decreasing with increasing remoteness [22]. Patients are more likely to travel long distances to access PM services with increasing distance from metropolitan areas. Australian women with prolapse have a right to equitable, high-quality services regardless of their geographical location.

Training in PM was highlighted among ten research priorities in a UK study in 2018 [30]. Subsequently, Dwyer et al [20], in their review of HCP training in PM, concluded that “a standardised approach to pessary practitioner training is advocated to ensure that women receive safe, evidence-based pessary care”. The current study revealed that HCPs had varied training in PM, including 11 (2%) with no training and most without workplace competency standards. The majority of HCPs wanted further training in PM, including all respondents in remote locations, where none had completed postgraduate training in PM. Our study suggests a need for training standards and accessible training programs, in line with the desire for further training, expressed especially by rural/remote HCPs.

Consistent with international practice, ring pessaries were most commonly used [13, 15]. In the current study, silicone rings were most frequently used, compared with the UK where PVC and vinyl rings were common [13]. Shelf and cube pessaries were the more common alternative to rings in the UK [13] and France [15] respectively.

Physiotherapists were fitting pessaries in a younger (< 60 years) population of Australian women compared to doctors and nurses. This contrasts with international reports of women over 50 [15] or 60 years [12] being fitted most frequently with pessaries. We speculate that many physiotherapists in our survey are treating postpartum women, and are encouraging them to return to physical activity using a pessary if they have symptomatic POP. This warrants further investigation.

A major strength of this study was the high number of responses from urogynaecologists and physiotherapists and the broad sampling of HCPs involved in PM. As of September 2022, the Australian Health Practitioner Regulation Agency [26] had 36 registered urogynecologists, and 28 (78%) responded to our survey. The high number of responding physiotherapists allowed us to identify a strong trend in physiotherapy involvement in PM not previously reported.

There were several limitations to our study. Direct targeting of members of professional associations was possible at no cost to us for GPs, nurses, urogynaecologists and physiotherapists, but not for obstetricians and gynaecologists. To mitigate this, we implemented purposive and snowball sampling techniques using alternative databases, professional contacts and internet hand-searching for these professional groups. Despite this, obstetrician–gynaecologists remained under-represented in our results. We did not consider urologists as providers of PM initially and omitted to target the Urological Society of Australia and New Zealand, leading to probable under-representation of this professional group. Being physiotherapy researchers, snowball sampling of physiotherapists may have been more successful, potentially skewing our data, as discussed. The survey allowed respondents to select all potential training options, regardless of profession. Some responses may have been erroneous, specifically two physiotherapists who reported completing fellowship training, and an additional ten physiotherapists and seven doctors who reported undergraduate training in PM, which, to our knowledge, is not available in Australia. These were included in our data set, but excluded from analyses pertaining to training in PM. Finally, as our study was performed in Australia, we acknowledge that the differences in the structure and geographical distribution of health services in Australia may limit the generalisability of our results internationally.

Women all over Australia are affected by symptomatic POP. A pessary can provide low-cost, effective symptom relief, allowing many women with POP to lead full and active lives again [10]. Our study highlights the difficulty women may experience accessing high-quality pessary services with adequately trained HCPs, and with governance structures ensuring safe, evidence-based care. Our results highlight the need to address the educational needs of HCPs across all disciplines, with the possible exception of urogynaecologists. A range of learning modalities should be considered to provide accessible and flexible learning opportunities with agreed standards and competency-based assessment. A model for multi-disciplinary pessary training is currently under development in the UK, which may have limited application in Australia due to the differences in health care delivery in the two countries. Future research will need to investigate optimal pessary training methods.

## Conclusion

Our survey of Australian HCPs revealed a range of professional groups providing PM. It is the first to report large numbers of physiotherapists providing PM, predominantly for younger women. Urogynaecologists and nurses, however, were the professions fitting the greatest number of pessaries per month. HCPs had variable training and experience in PM, including some who had no training at all. This study highlights the need for accessible pessary services for Australian women with POP, standardised and competency-based training opportunities for pessary care providers and governance structures ensuring safe and evidence-based care.

### Supplementary information

ESM 1 Questionnaire (DOCX 55.2 kb)
