# Supplementary material for: Pessary management practices for pelvic organ prolapse among Australian health care practitioners: a cross-sectional study
Source: Int Urogynecol J. 2023 May 24;34(10):2519–27. doi: 10.1007/s00192-023-05540-2 (PMC10590327; doi:10.1007/s00192-023-05540-2)
Supplement: Supplementary file 1 — Questionnaire (DOCX 55.2 kb) [file 192_2023_5540_MOESM1_ESM.docx]

**Appendix 1: Questionnaire**


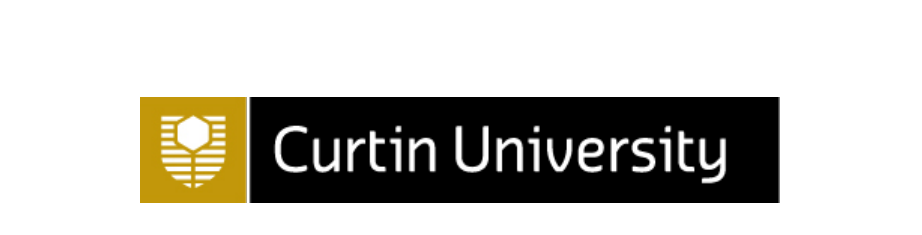


**A survey of pessary management practices for pelvic organ** **prolapse amongst Australian health care practitioners.**
   *Thank you for completing this survey if you are involved in pessary management. The survey should only take 5 minutes of your time. Please only complete it once. All questions require a response. Incomplete surveys will be recorded. You can save and resume your response until the survey closes. The survey will close on the 31st of August 2022.*

Please see attached the [Participant Information Statement](https://curtin.au1.qualtrics.com/CP/File.php?F=F_3xuL0H9iZ06AHoG) for further details about the study.

I have received information regarding this research and had an opportunity to ask questions. I believe I understand the purpose, extent and possible risks of my involvement in this project, and I voluntarily consent to take part.

- I consent

Q1. What is your profession?

*Medical Practitioner*

- Urogynaecologist
- Gynaecologist
- Obstetrician
- Obstetrician-gynaecologist
- GP Obstetrician
- GP

*Nurse*

- Nurse practitioner
- Clinical Nurse
- Registered Nurse
- Registered Nurse / Registered Midwife

*Physiotherapist*

- Undergraduate qualification (this includes graduate entry master’s program)
- Postgraduate qualification

*Other*

- Please specify your profession in the text box:

Q2. How long (in years) have you been qualified in your profession?

Q3. How long (in years) have you been providing pessary management services?

Q4. What state/territory do you work in? (Select all that apply)

- Australian Capital Territory
- New South Wales
- Northern Territory
- Queensland
- South Australia
- Tasmania
- Victoria
- Western Australia

Q5. What geographical area do you work in? (Select all that apply)
MM categories refers to the Modified Monash Model, details of which can be found [here](https://www.health.gov.au/sites/default/files/documents/2020/07/modified-monash-model-fact-sheet.pdf).

- Metropolitan (Major Australian Cities accounting for 70% of Australia's Population) (MM1)
- Regional (Inner and Outer) Centres- >50,000 residents (MM2)
- Large Rural Towns -15,000-50,000 residents (MM3)
- Medium Rural Towns- 5,000-15,000 residents (MM4)
- Small Rural Towns- All remaining inner/outer regional areas- 1,000-5000 residents (MM5)
- Remote Communities- Mainland communities
- Very remote areas- all other communities or islands >5km offshore (MM7)

Q6. Please list the postcode(s) of your workplace(s). Please only list those where pessary management is part of your role, separating each postcode with a comma.

Q7. Do you agree that you currently provide pessary management for patients who travel a long distance to access your service?

- Yes
- No

Q8. In which clinical setting(s) do you currently fit pessaries in? (Select all that apply)

*Public Hospital*

- Inpatient
- Pessary clinic
- Outpatient clinic
- Community clinic
- Outreach/rural clinic

*Private Hospital*

- Pessary clinic
- Outpatient clinic

*Private practice*

- Sole practitioner private practice
- Single profession private practice with only one pessary provider
- Single profession private practice with more than one pessary provider
- Multidisciplinary private practice with only one pessary provider
- Multidisciplinary private practice with more than one pessary provider

*Other*

- Please specify your clinical setting in the text box:

Q9. Do you provide pessary management as part of a shared-care arrangement with any other health professional? (Select all that apply)

- No
- Yes - with urogynaecologist(s)
- Yes - with gynaecologist(s)
- Yes - with general practitioner(s)
- Yes - with nurse(s)
- Yes - with physiotherapist(s)
- Other (please specify in text box)

Q10. What training have you undertaken in pessary management? (Select all that apply)
Fellowship training

- Undergraduate training
- Post graduate university qualification
- Non-university based professional development course(s)
- Mentoring / on the job training
- None
- Other (please specify in text box)

Q11. Do you provide mentoring or support to any other professionals who provide pessary management?
Yes

- No

If Q11. Do you provide mentoring or support to any other professionals who provide pessary Q12. To whom do you provide mentoring for pessary management? (Select all that apply)
Others from your own profession

- Others from different professions

Q13. Do you receive mentoring or support from any other professionals who provide pessary management?
Yes

- No

Q14. From whom do you receive mentoring for pessary management? (Select all that apply)

- Others from your own profession
- Others from different professions

Q15. Does your workplace require you to meet a pessary competency standard to provide pessary management?

- Yes
- No

Q16. What is the **most common** age group that you provide pessary management for?

- <40 years
- 40-60 years
- > 60 years

Q17. Which aspects of pessary management do you provide? (Select all that apply)

- Pessary sizing & fitting
- Follow-up and changing pessaries
- Teaching patient self-management
- Other (please specify in the text box)

Q18. Which pessaries do you use for prolapse management? (Select all that apply)
Ring (vinyl or PVC)

- Ring (silicone)
- Ring with support
- Cube
- Gellhorn
- Dish
- Donut
- Shelf
- Shaatz
- Hodge
- C-POP
- Combination of 2+ pessaries simultaneously
- Other (please specify in text box)

*The following three questions relate to how commonly you fit different pessary types.*

Q19. What is the **most common** pessary type that you fit for management of prolapse?

- Ring pessaries
- Cube
- Gellhorn
- Dish
- Donut
- Shelf
- Shaatz
- Hodge
- C-POP
- Combination of 2+ pessaries simultaneously
- Other (please specify in text box)

Q20. What is the **second most** common pessary type that you fit for management of prolapse?

- Ring pessaries
- Cube
- Gellhorn
- Dish
- Donut
- Shelf
- Shaatz
- Hodge
- C-POP
- Combination of 2+ pessaries simultaneously
- I do not fit another type of pessary
- Other (please specify in text box)

Q21. What is the **third most** common pessary type that you fit for management of prolapse?

- Ring pessaries
- Cube
- Gellhorn
- Dish
- Donut
- Shelf
- Shaatz
- Hodge
- C-POP
- Combination of 2+ pessaries simultaneously
- I do not fit another type of pessary
- Other (please specify in text box)

Q22. On average, how many pessaries do you fit per month?

Q23. Would you like to undertake further training in pessary management?

- Yes
- No

ki End of Survey If Q23. Would you like to undertake further training in pessary management?

Q24. If you would like further training in pessary management, what areas would you like further training in? (Select all that apply)
Management of adverse events

- Management of complex cases
- Risk mitigation
- Sizing and fitting of pessaries (please specify pessary type in text box)
- Other (please specify in text box)
